# Molecular characterization of gut microbiome in weaning pigs supplemented with multi-strain probiotics using metagenomic, culturomic, and metabolomic approaches

**DOI:** 10.1186/s42523-022-00212-w

**Published:** 2022-11-24

**Authors:** Woong Ji Lee, Sangdon Ryu, An Na Kang, Minho Song, Minhye Shin, Sangnam Oh, Younghoon Kim

**Affiliations:** 1 Department of Agricultural Biotechnology and Research Institute of Agriculture and Life Science, Seoul National University, Seoul, 08826 Korea; 2 Division of Animal and Dairy Science, Chungnam National University, Daejeon, 34134 Korea; 3 Department of Microbiology, College of Medicine, Inha University, Incheon, 22212 Korea; 4 Department of Functional Food and Biotechnology, Jeonju University, Jeonju, 55069 Korea

**Keywords:** Multi-strain probiotics, Multiomics, Gut microbiome, Metabolite, Weaning pigs

## Abstract

**Background:**

Probiotics have been reported to exhibit positive effects on host health, including improved intestinal barrier function, preventing pathogenic infection, and promoting nutrient digestion efficiency. These internal changes are reflected to the fecal microbiota composition and, bacterial metabolites production. In accordance, the application of probiotics has been broadened to industrial animals, including swine, which makes people to pursue better knowledge of the correlation between changes in the fecal microbiota and metabolites. Therefore, this study evaluated the effect of multi-strain probiotics (MSP) supplementation to piglets utilizing multiomics analytical approaches including metagenomics, culturomics, and metabolomics.

**Results:**

Six-week-old piglets were supplemented with MSP composed of *Lactobacillus* isolated from the feces of healthy piglets. To examine the effect of MSP supplement, piglets of the same age were selected and divided into two groups; one with MSP supplement (MSP group) and the other one without MSP supplement (Control group). MSP feeding altered the composition of the fecal microbiota, as demonstrated by metagenomics analysis. The abundance of commensal *Lactobacillus* was increased by 2.39%, while *Clostridium* was decreased, which revealed the similar pattern to the culturomic approach. Next, we investigated the microbial metabolite profiles, specifically SCFAs using HPLC–MS/MS and others using GC–MS, respectively. MSP supplement elevated the abundance of amino acids, including valine, isoleucine and proline as well as the concentration of acetic acid. According to the correlation analyses, these alterations were found out to be crucial in energy synthesizing metabolism, such as branched-chain amino acid (BCAA) metabolism and coenzyme A biosynthesis. Furthermore, we isolated commensal *Lactobacillus* strains enriched by MSP supplement, and analyzed the metabolites and evaluated the functional improvement, related to tight junction from intestinal porcine enterocyte cell line (IPEC-J2).

**Conclusions:**

In conclusion, MSP administration to piglets altered their fecal microbiota, by enriching commensal *Lactobacillus* strains. This change contributed amino acid, acetic acid, and BCAA concentrations to be increased, and energy metabolism pathway was also increased at in vivo and in vitro. These changes produced by MSP supplement suggests the correlation between the various physiological energy metabolism functions induced by health-promoting *Lactobacillus* and the growth performance of piglets.

**Supplementary Information:**

The online version contains supplementary material available at 10.1186/s42523-022-00212-w.

## Background

As pork consumption has been gradually increased in the worldwide, the pig industry has also been expanded but issues with pig sanitation and disease management have emerged on one side. In the past, the antibiotics were generally administrated to treat animal diseases or infections to enhance survival rates and promote animal production [1]. As food safety has become an increasingly serious international problem, antibiotic resistance gene propagation has been considered as a growing public concern in this decade, so the use of antibiotics as a feed additive for livestock was decided outlawed [2], limiting the use of growth promoters (AGPs). Therefore, numerous studies have been carried out to explore alternatives that are less likely to induce antibiotic resistance, while retaining food safety, preservation, and efficiency, in order to replace antibiotics [3, 4]. According to the previous studies, using probiotics as an alternative to low-dose antibiotics, was significantly safer, and the flavor of the animal product was not impacted when the probiotics survived and colonized in the gastrointestinal tract. These features were resulted in a substantial increase in the interest in probiotics applications in the animal industry [5, 6]. To be beneficial to the host, probiotics must withstand the severe conditions of the gastrointestinal system. Using transmembrane proteins and extracellular matrix components, probiotics can colonized to the surface of the host intestine, consequently enhancing the eradication of pathogens [7, 8]. In addition to preventing pathogen adhesion by competitive exclusion, probiotics influence the hosts immune response, contribute to the integrity of the intestinal wall barrier, and produce substances such as bacteriocins, organic acids, and metabolites that can inhibit pathobiont growth [9, 10]. In swine industry, probiotics have been employing in all phases of pig production (from early weaned piglets to growing-finishing piglets) [11]. Especially, the administration of *Lactobacilli, Bacillus*, which were frequently used as probiotics to piglets increased growth performance and enhanced gut immune system [12–14].

The mammalian gastrointestinal tract (GIT) is habitat to a highly complex and diverse microbial population that interacts with the host in mutualistic manner, contributing to host growth and health by avoiding gut microbial dysbiosis [15]. A microbiome that is considered to be “healthy” offers essential molecular signals to the immune system in forms of microbial surface antigens and metabolites. These molecular cues are necessary for the maturation of immunological tissues and the fine-tuning of immune responses. The development of high-throughput DNA sequencing technologies permitted the immediate classification of samples without the need for cultivation. These technical advancements provided a reliable tool for characterizing complex microbial communities occurring in various habitats and analyzing changes in the communities structure over time [15], unfortunately, more than a century of microbiology research, determining the microbial diversity of a specific habitat still remains as a challenge. One of the difficulties was that scientists generally estimate a range between 10^7^ and 10^12^ species of bacteria per 1 g of stool, but few species have been isolated by practical culture in lab scale [16, 17]. Moreover, the composition of an individual microbiome varied significantly and the difference grows substantially over time within an individual. Nonetheless, fundamental common characteristics exist in the microbial communities occupied in the host GIT [18], which makes multiomics approaches, such as metagenomics, metabolomics, and culturomics have been considered to be adequate to study microbial populations. With the perception of multiomics, many studies have been establishing a correlation between the dynamics and diversity of the microbiome and health and disease successfully [19].

The earliest method isolating bacteria was a culture method, however, due to the advances in sequencing techniques, notably metagenomics, it is the fact that the culture method has been losing its status [20]. The genomic databases are insufficient to fully assign an exact taxonomic categorization to a large number of sequences. Furthermore, metagenomics analysis cannot provide a pure culture of microorganisms, which is necessary for the strain characterization, and host-microbiota interaction studies, while the improvements in culturomics could allow a spectrum of microorganisms if appropriate set-ups and equipments are accessible. The technical advancements was provided the potential clue to the cultivation of “uncultivable organism” challenges [20]. Culturomics has been played an important role in the description of the microbiota, particularly in filling metagenomics gaps by newly isolated bacterial species in humans and animals [21, 22].

Although methods based on mass spectrometry and chromatography have been employed for more than a century, limited efforts have been applied to investigations of the relationship between the host and the fecal microbiota lately [23–25]. Targeted and untargeted metabolomic and metaproteomic approaches have the ability to investigate the chemical diversity and biochemical potential of synthetic and natural microbial communities. Short-chain fatty acids (SCFAs), such as acetic, propionic, and butyric acids, which are the primary bacterial metabolites in the gut, function as signal molecules to inhibit histone deacetylation and exert favorable effects on the homeostasis of carbohydrate and lipid metabolism, gastrointestinal motility, and immunity. This is accomplished by directly binding to their specific G-protein-coupled receptors (GPR) 41 and GPR43 [26, 27]. The carbohydrate induced SCFA such as rectal butyric acid treatment significantly enhanced intestinal motility and alleviated stomach pain or discomfort [27]. In addition, prior research indicated that carbohydrates, sugar alcohols, bile acids, carboxylic acids, and secondary bile acids were able to influence the colonization of commensals in the digestive system [28]. Metabolomics and proteomics analysis will continue to expand the understandings of the many impacts of the microbiome on host physiology, suggesting potentials for the development and evaluation of diagnostics and therapies. In this study, we investigated the molecular effect of MSP supplementation on the piglet gut microbiome, and isolated *Lactobacillus* which were enriched by MSP was subjected to further researches to determine the effect of MSP supplement.

## Results

### Growth performance and populations of gut microbiota

Initially, we observed the changes of growth performance with or without MSP supplementation of piglets. Similar to the previous report [29], there was no dramatic difference in results of growth performance including body weight, feed intake, average daily feed intake (ADFI), and gain-to-feed ratio (G:F) between control and MSP groups during 6 weeks (data not shown). Despite an increasing trend in body weight (25.28 vs. 26.54 kg; *p* > 0.05) and feed intake (27.62 vs. 29.01 kg; *p* > 0.05) in piglets fed MSP, still, weaning pigs fed with MSP had significantly improved ADG (455.23 vs. 491.02 g/d; *p* < 0.05).

**Fig. 1:**
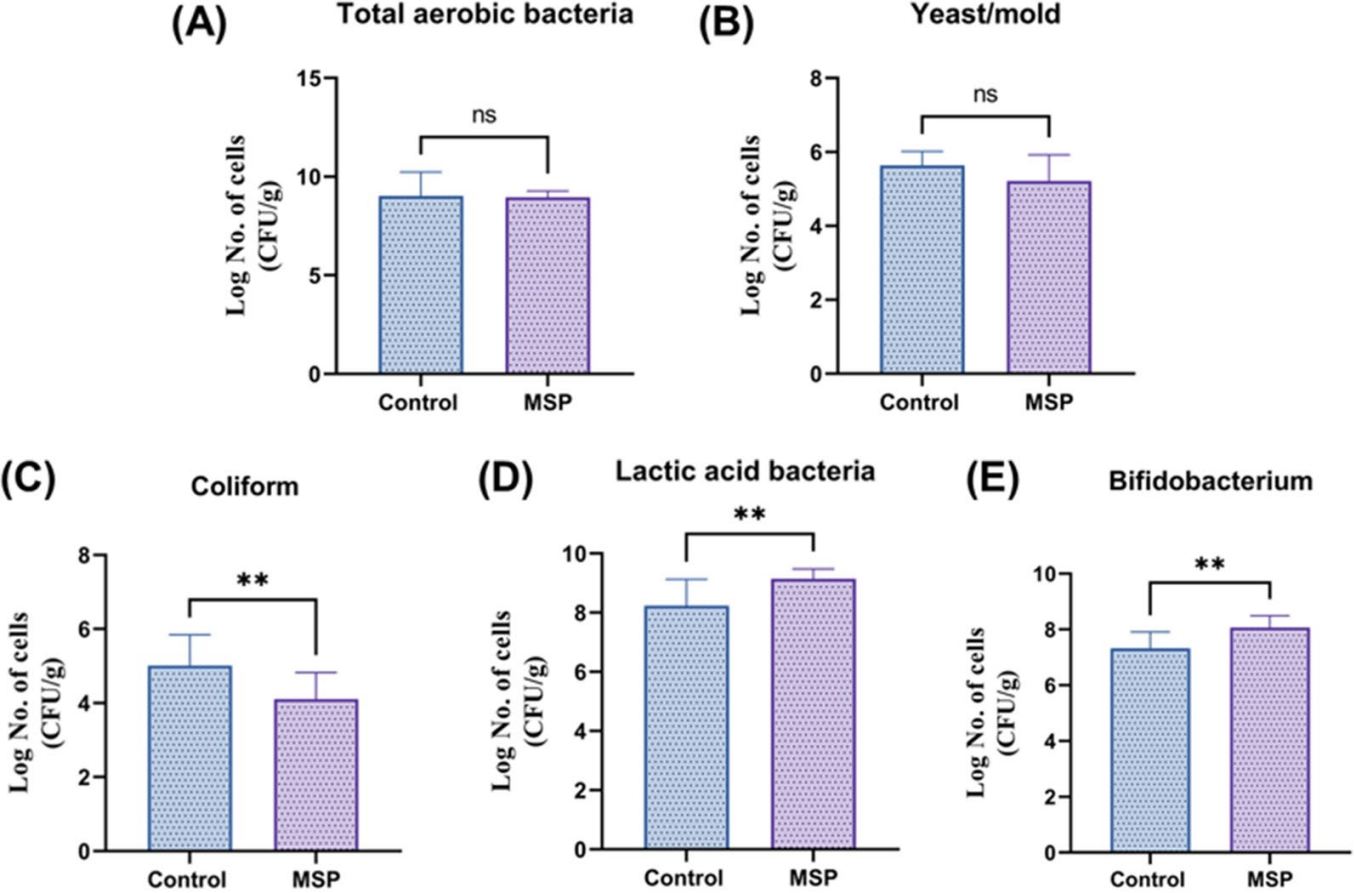
Comparison of microbial population of piglet feces within MSP and control group. **A** Quantitative analysis of total aerobic bacteria between MSP and control groups, **B** yeast/mold, **C** coliform, **D** lactic acid bacteria and **E** *Bifidobacterium* (**p < 0.01)

In addition, plate counts were performed to explore the abundance in lactic acid bacteria (LAB), *Bifidobacterium*, and coliforms as well as total aerobic bacteria and yeast/mold in the control and MSP groups (Fig. 1). Interestingly, the number of LAB and *Bifidobacterium* in fecal samples were significantly increased in the MSP group compared to the control group (*p* < 0.05) (Fig. 1D, E), whereas the population of coliform was decreased by MSP supplementation (Fig. 1C). Importantly, previous studies and our colleagues indicated that pigs supplemented with specific probiotics exhibited improved nutrient digestibility and meat quality coupled with the changes of gut microbiota composition and immune response [30–33]. Therefore, the potentiality has been suggested that growth performance would be increased due to the alterations of microbiota compositions induced by MSP supplementation.

### 16 S rRNA based fecal microbiota profiles in MSP and control piglets

**Fig. 2:**
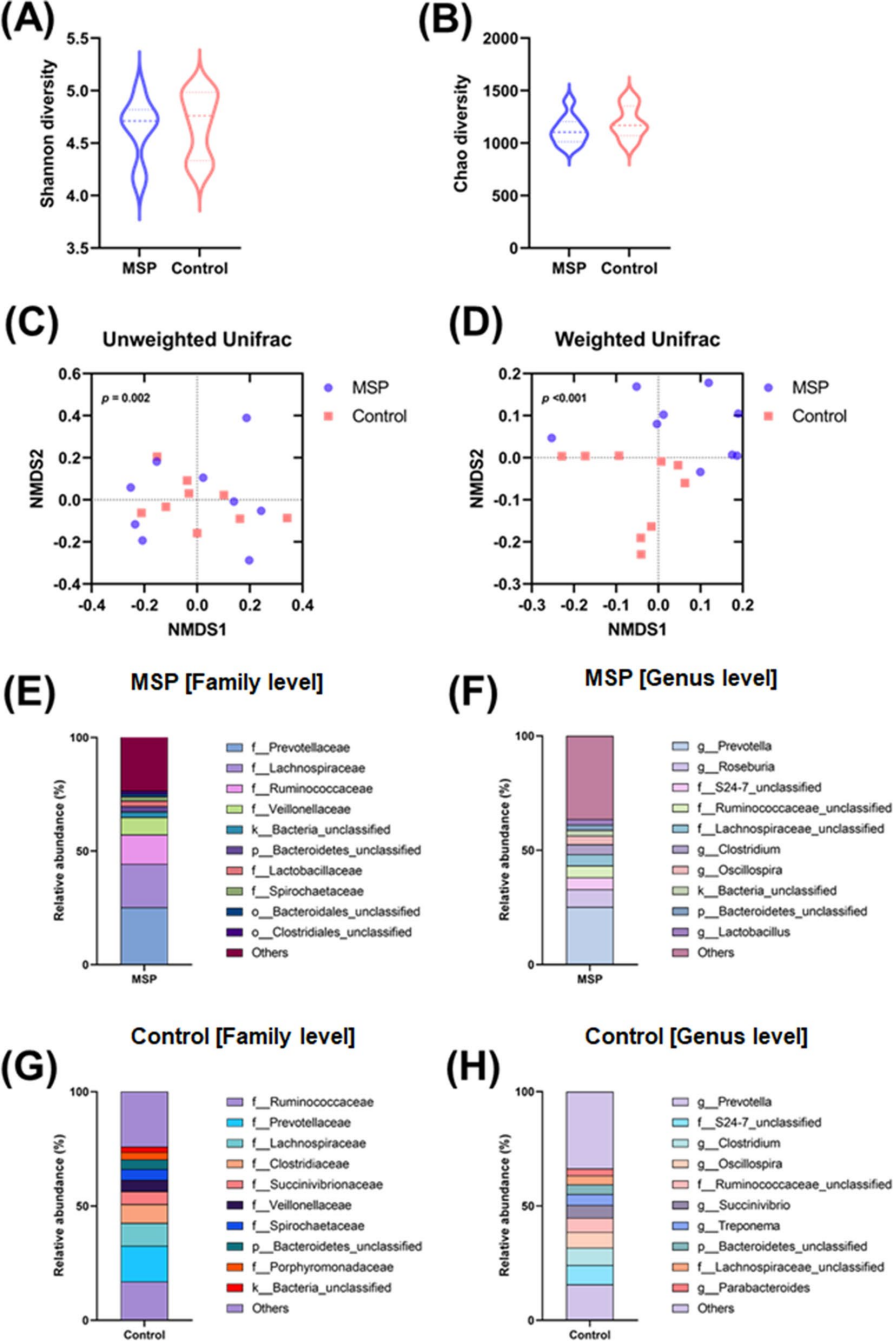
Microbial Characterization of piglet fecal microbiota utilizing metagenomics. Differences in microbial communities in MSP and control group at 6 weeks (n = 9 per group). Violin plots reflect differences in bacterial diversity in fecal microbiota according to the Shannon index and Chao richness. **A** Shannon diversity representing alpha diversity and **B** Chao diversity. **C**, **D** Nonmetric multidimensional scaling analysis of weighted and unweighted UniFrac distance. Relative abundance of 10 dominant OTUs in MSP group piglet fecal microbiota at family level **E** and genus level **F**. Relative abundance of 10 dominant OTUs in control group piglet fecal microbiota at family level **G** and genus level **H**

The precise effects of probiotics in modulating pig microbiota and growth performance still remains unclear over a decade. Here, this study was aimed to identify the role of MSP in dynamics of gut microbiota in weaned pigs through multiomics analysis. The V4 hypervariable region of the 16 S rRNA gene was sequenced to identify the fecal microbiota composition and diversity in MSP and control piglets. Total 1,197,453 sequences were acquired and were assigned to 2,318 OTUs based on 97% sequence similarity. In the alpha diversity analysis, which evaluated the diversity within piglet fecal microbiota, the Chao and Shannon indices were calculated to assess the diversity and richness of the fecal microbiota in MSP and control piglets. Shannon and Chao diversity index indicated no significant difference between the MSP and control group (MSP vs. control: 4.63 ± 0.29 vs. 4.67 ± 0.31 and 1119.16 ± 137.45 vs. 1196.86 ± 157.01, *p* > 0.05) (Fig. 2A, B). Then, to examine the similarity or dissimilarity of the microbial community between the MSP and control groups, beta diversity was assessed (Fig. 2C, D). The microbial ecology of each piglet is represented by each point in the NMDS (non-metric multidimensional scaling), with closer distances between points indicating more comparable microbial ecology. Using analysis of similarity (ANOSIM), the unweighted UniFrac distance and the weighted UniFrac distance showed substantial discrepancies. The beta diversity results showed the diversification that emerges in the fecal microbiota as a result of supplemental MSP. These results contribute to alterations in the compositional heterogeneity of fecal microbiota as a result of MSP supplementation.

In MSP group, Prevotellaceae, Lachnospiraceae, Ruminococcaceae and Veillonellaceae were dominant family classification, while the control group was Ruminococcaceae, Prevotellaceae, Lachnospiraceae, Clostridiaceae and Succinivibrionaceae (Fig. 2E, G). At the genus level, *Prevotella* was dominant in both MSP (25.22%) and control groups (15.68%). Notably, MSP supplement increased *Lactobacillus* to 2.39% while the relative abundance of *Clostridium* taxa, which are associated with a variety of pathogens, was significantly higher in the control group (control, 7.58%; MSP, 4.3%; *p =* 0.0236) (Fig. 2F, H).

To assess whether the effect of MSP feeding could be different depending on the growth phases of piglets, the fecal microbiota of piglets in the early, middle and late phase were compared and analyzed (Additional file 1: Fig. S1). Unfortunately, significant changes in fecal microbiota between the growth phases were not observed except the Shannon index. Although there was no remarkable alternation, relative abundance of *Lactobacillus* was obviously increased as a result of MSP feeding among all growth phases. These results indicated that the MSP supplement made substantial alterations to fecal microbiota of piglet. Since *Lactobacillus* has been generally regarded as a commensal bacterium, it has been deserved of research on the cascade impacts on piglet gut microbiome.

#### Microbiological characterization of fecal microbiota in piglets supplemented with MSP through comparison between culturomic and metagenomic approaches

**Fig. 3:**
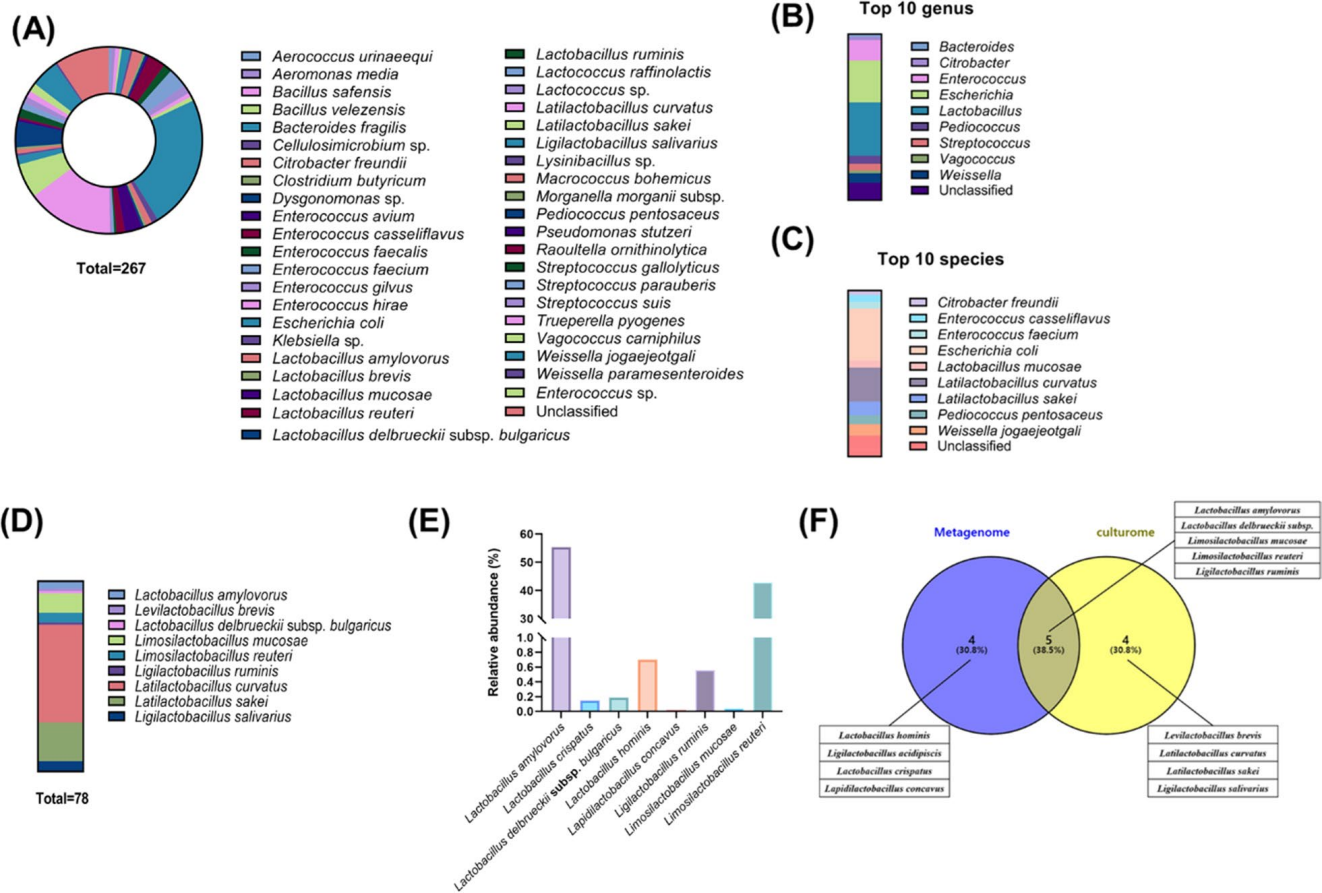
Microbial characterization of MSP group utilizing culturomics and comparison analysis with metagenomics. **A** Identification of MSP group piglet fecal microbiota through culturomics approaches. **B** 10 dominant bacteria genus of MSP piglet fecal microbiota in culturomics approaches. **C** bacteria species. **D** Composition of *Lactobacillus* spp. detected from MSP piglet fecal microbiota through culturomics approaches. **E** Relative abundance of OTUs identifying *Lactobacillus* spp. **F** Venn diagram depicting the number of bacterial species identified by culturomics (yellow) and metagenomics (purple), along with the proportion of species detected by both approaches (gray)

According to the previous results, MSP supplementation increased *Lactobacillus* abundance in metagenomics analysis, which presented the necessity about further characterization of *Lactobacillus* compositional alterations. Therefore, we applied culturomics to investigate whether the metagenomics matched with culturable isolates from MSP piglet feces and identified the *Lactobacillus* species which were increased by MSP supplement. Total 267 bacterial colonies were isolated; 42 species were classified and 9% were unidentified (Fig. 3A). 42 species were partitioned into 23 genera, the dominant genera were *Lactobacillus* (29.59%), *Escherichia* (23.22%) and *Enterococcus* (11.24%) (Fig. 3B). In *Lactobacillus*, 9 species were classified and the dominant species were *L. curvatus* (51.28%), *L. sakei* (20.51%) and *L. mucosae* (10.26%). These species were included in 10 dominant species in which total bacteria was isolated from piglet feces (Fig. 3C, D). Notably, *Lactobacillus* species isolated from culturomics were matched with the metagenome relative abundance by 38.5%, still, *Lactobacillus* species composing MSP were not regarded as dominant in both analyses (Fig. 3D–F). These results revealed that MSP supplement increased *Lactobacillus* as commensal in gut environment other than those included in MSP in piglet fecal microbiota, and these commensal *Lactobacillus* strains would improve growth performance or influence a particular metabolic pathway.

#### Metabolomic profile of MSP supplementation in piglets

**Fig. 4:**
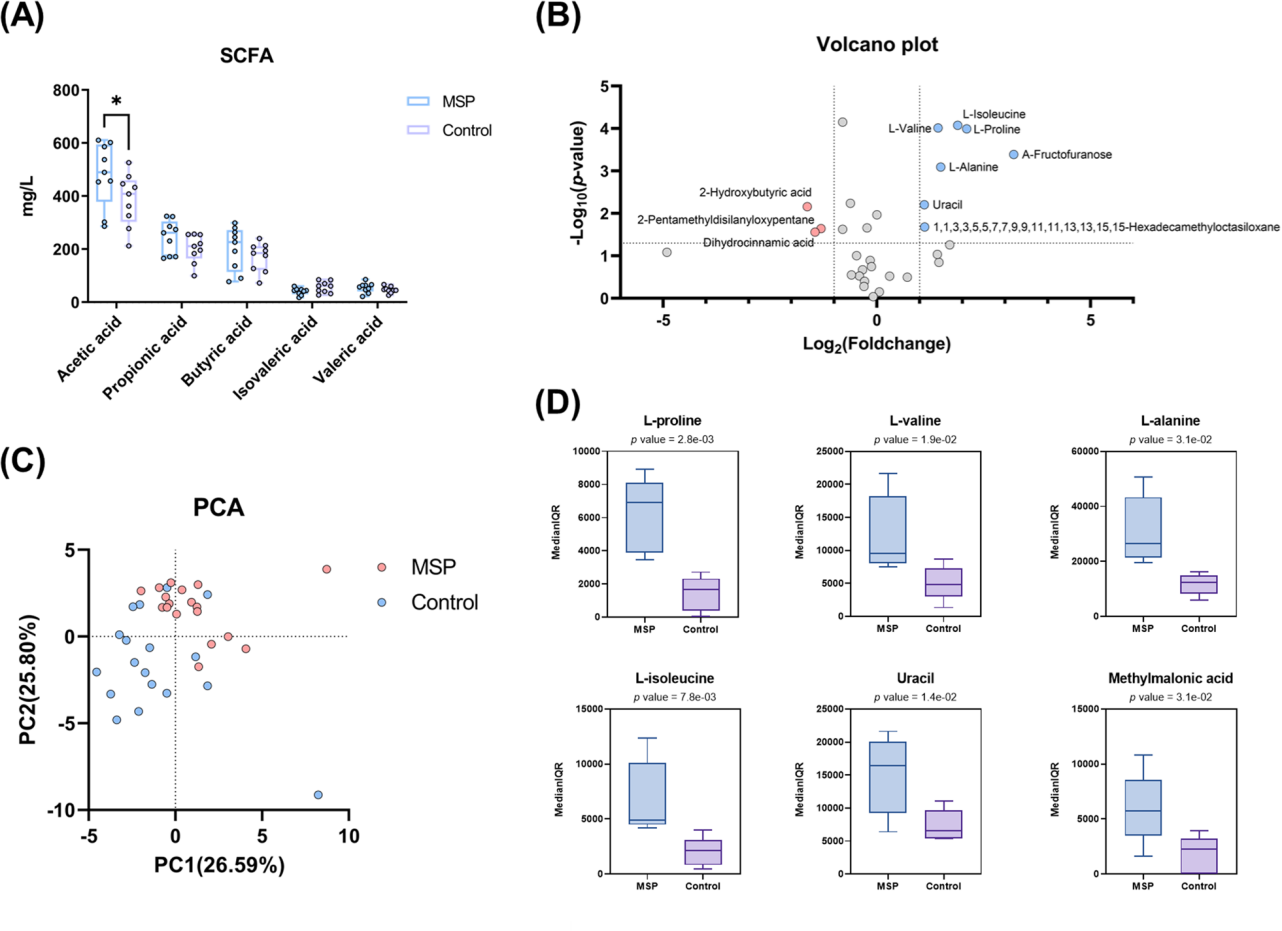
Difference concentration and variation of SCFAs and metabolites compared with MSP and control piglets. Metabolomic analysis of piglet feces at 6 weeks. **A** SCFA profiling using HPLC–MS/MS. **B** The results of the volcano plot analysis showed that 7 peak pairs (FC > 1, *p ≤* 0.05) were increased, whereas 3 peak pairs (FC < 1, *p* ≤ 0.05) were reduced. All metabolites that were positively and putatively identified with high confidence were matched using the NIST library. **C** Principal component analysis (PCA) score plots of metabolites in the feces of piglets between the MSP and control groups. **D** Selected metabolite and bacterial boxplots showed differences between the MSP and control groups. Mean (SD) values of each group are provided by Student’s *t* test. Median (IQR) values are provided by the Mann–Whitney U test

Our metagenomic and culturomic results indicated the potential of MSP altering piglet fecal microbiota. However, since the effects of MSP supplement were still uncertain, we decided to analyze microbial metabolite profiles, focusing on SCFAs and other metabolites, using HPLC-MS/MS and GC-MS, respectively. Each peak represents a specific index and was determined by comparing the corresponding retention time and peak mass (*m/z* values). For further metabolomic analysis consisting of metabolic pathways and metabolites enrichment, 37 most intense chromatographic peaks were selected. The MSP-supplemented piglets had significantly higher acetic acid concentration (*p* = 0.02) and propionic and butyric acid concentrations were not significant but tended to be higher, while isovaleric acid and valeric acid in the MSP and control groups were not significantly different (Fig. 4A). The volcano plot and the boxplots represented l-alanine, l-valine, l-isoleucine, l-proline, uracil, and A-fructofuranose expression levels were all upregulated in MSP groups (log_2_ fold change > 1, − log_10_ *p* > 1.3) (Fig. 4B, D). The metabolic profiles PCA score plots revealed a clear distinction between piglets supplemented with MSP and control (Fig. 4C). Taken together, MSP supplement increased commensal *Lactobacillus* in piglet fecal microbiota and elevated the concentration of acetic acid, proline, uracil and BCAA such as valine, isoleucine. Therefore, it could be expected that commensal *Lactobacillus* regulated gut metabolism related to nutrient digestibility or barrier function.

#### Insights from correlation analysis of the metabolic activity of the piglet fecal microbiota

**Fig. 5:**
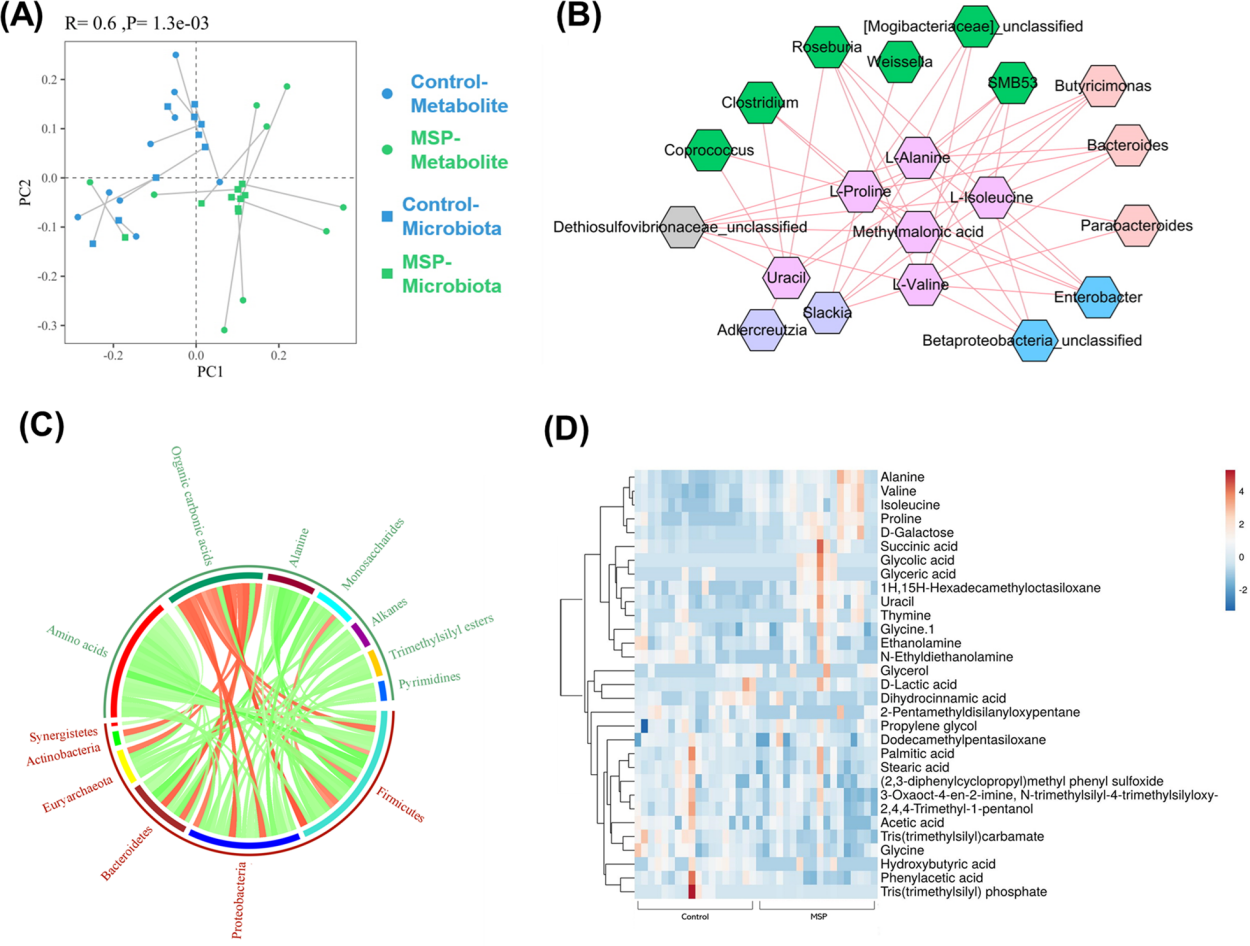
Correlation analysis between fecal microbiota and metabolites. The depiction of similarity analysis and SparCC correlation analysis results. **A** PA, the length of the lines connecting two locations reflects how well two datasets samples agree. The correlation coefficient R ranges from 0 to 1, and the closer it is to 1, the more similar the two datasets are. **B** Correlation between the functional metabolites and fecal microbiota. The pink nodes represent the functional metabolites and the other nodes of bacteria were grouped with different colors (functional false discovery rate (FDR) < 0.05). **C** SparCC correlations between bacteria and metabolites are depicted in a Circos plot. Red or green lines indicate positive or negative correlations. **D** Heatmap represent metabolites concentrations correlated with each group

To investigate the correlation within altered fecal metabolites, microbiota and physiological performance, we analyzed inter-relationship about the changes induced by MPS supplement. We employed SparCC (Sparse Correlations for Compositional data) correlation analysis among the results from multi-omics and the connection between metabolites (GC-MS metabolomics) and microbiota ecology (16 S rRNA gene amplicon sequencing) were analyzed using multi-omics dataset. Procrustes analysis (PA), superimposition and scaling of PCA models of two datasets, PA evaluated the congruence of two-dimensional data distributions. The overall similarity assessment between the MSP and control groups was statistically separated (*p* < 0.002) (Fig. 5A) and the correlation of them were represented as a network analysis. BCAA, uracil, and amino acids such as proline and alanine were tightly associated with fecal microbial population, notably Firmicutes, the phylum which *Lactobacillus* was included, was correlated with amino acid (Fig. 5B, C). Specifically, MSP supplement increased BCAA including valine, isoleucine and other amino acids such as alanine and proline in piglet feces (Fig. 5D). These results demonstrated the same context with the previous results that *Lactobacillus* were enriched by MSP supplement increased the concentration of BCAA.

**Fig. 6:**
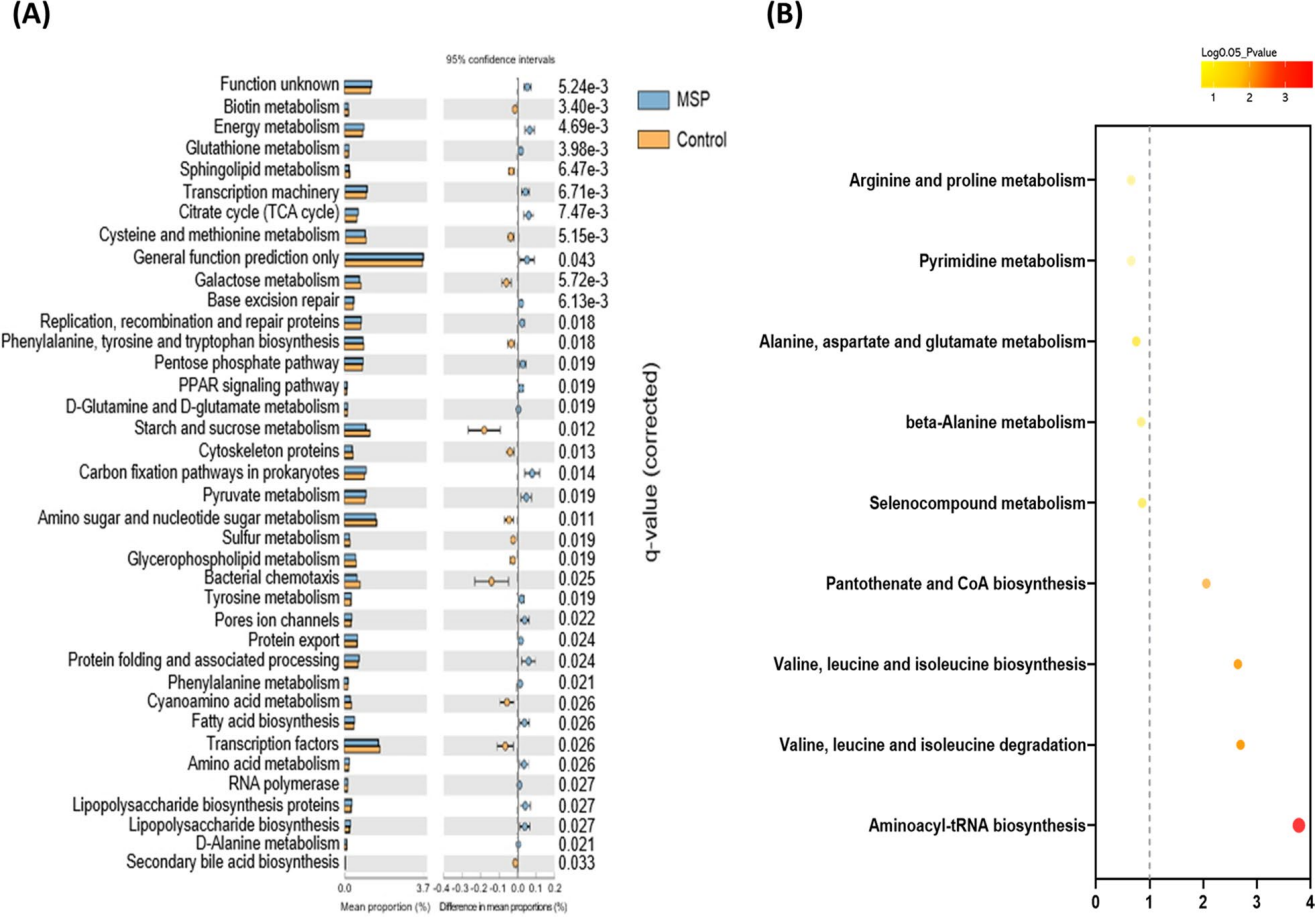
Metabolic pathway enrichment analysis was performed on differential metabolites. **A** KEGG pathways predicted in the fecal microbiota of the control and MSP groups using PICRUSt. Statistical analysis was carried out using STAMP software. **B** Differential metabolic pathways depicted up-regulated or down re-regulated metabolites involved in each metabolic pathway. *p-*value < 0.05 was considered statistically significant

The functional microbial pathways were analyzed using PICRUSt analysis and visualized using STAMP (Fig. 6A). The MSP supplement influenced energy metabolism including citrate cycle, pentose phosphate pathway and PPAR signaling pathway. The amino acid metabolism and aminoacyl-tRNA biosynthesis were also influenced in such ways of alanine, proline and BCAA (Fig. 6A, B). These results demonstrated that *Lactobacillus* enriched by MSP supplement increased BCAA and up-regulated energy and BCAA metabolism. On the basis of these findings, it is suggested that a comprehensive examination of the effects of the increased commensal *Lactobacillus* on the host as a consequence of MSP supplementation is necessary.

#### Functional analysis of ***Lactobacillus*** influenced by MSP supplement.

**Fig. 7:**
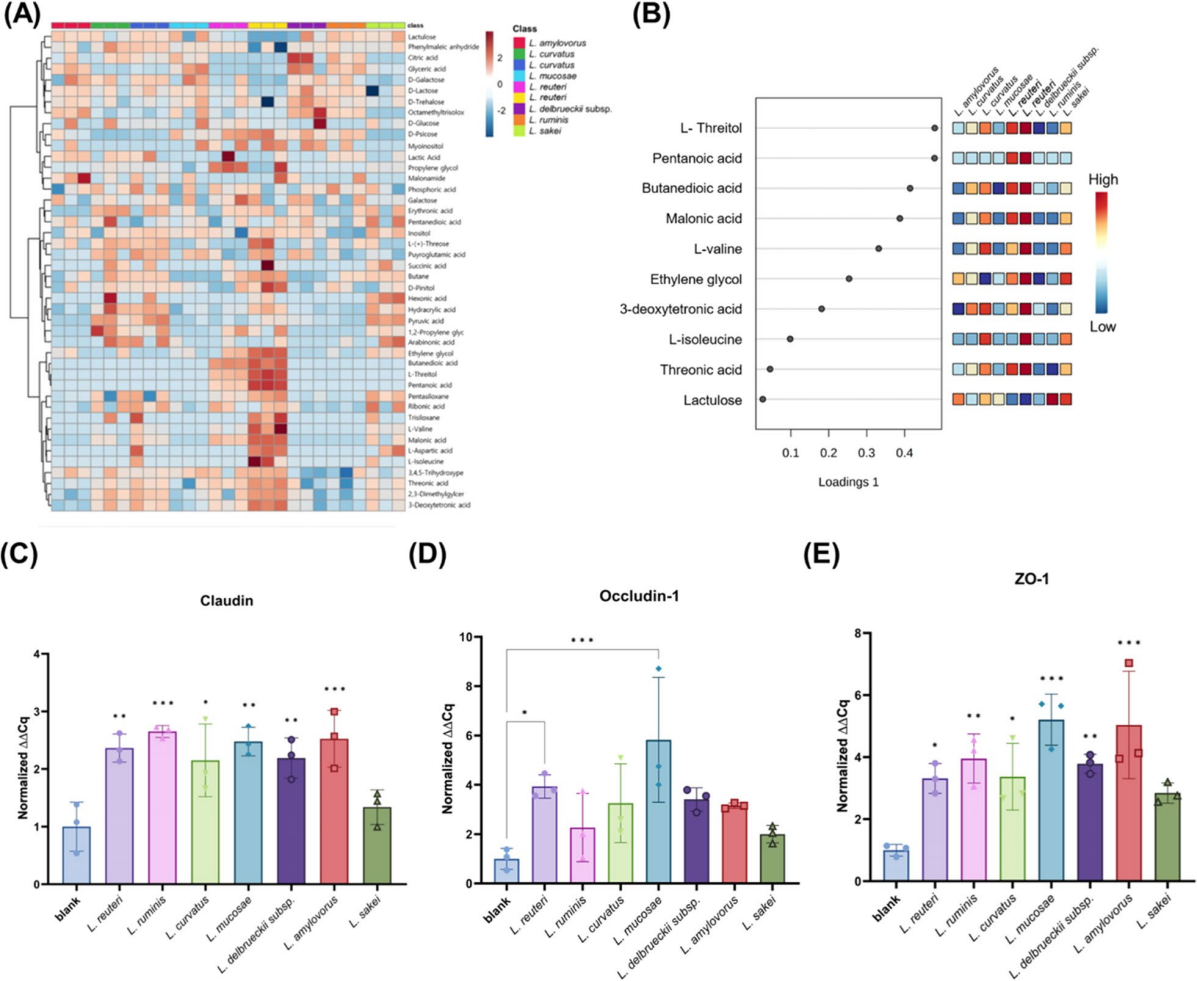
Functional analysis of commensal *Lactobacillus* strains enriched by MSP supplementation. **A** Heatmap provides intuitive visualization of metabolites. Each colored cell on the map corresponds to a concentration. **B** The sparse PLS-DA (sPLS-DA) algorithm represent selected metabolites for given component. Metabolites are ranked by the absolute values of their loadings. **C**–**E** Tight junction gene expression of enriched commensal *Lactobacillus* strains by MSP

**Fig. 8:**
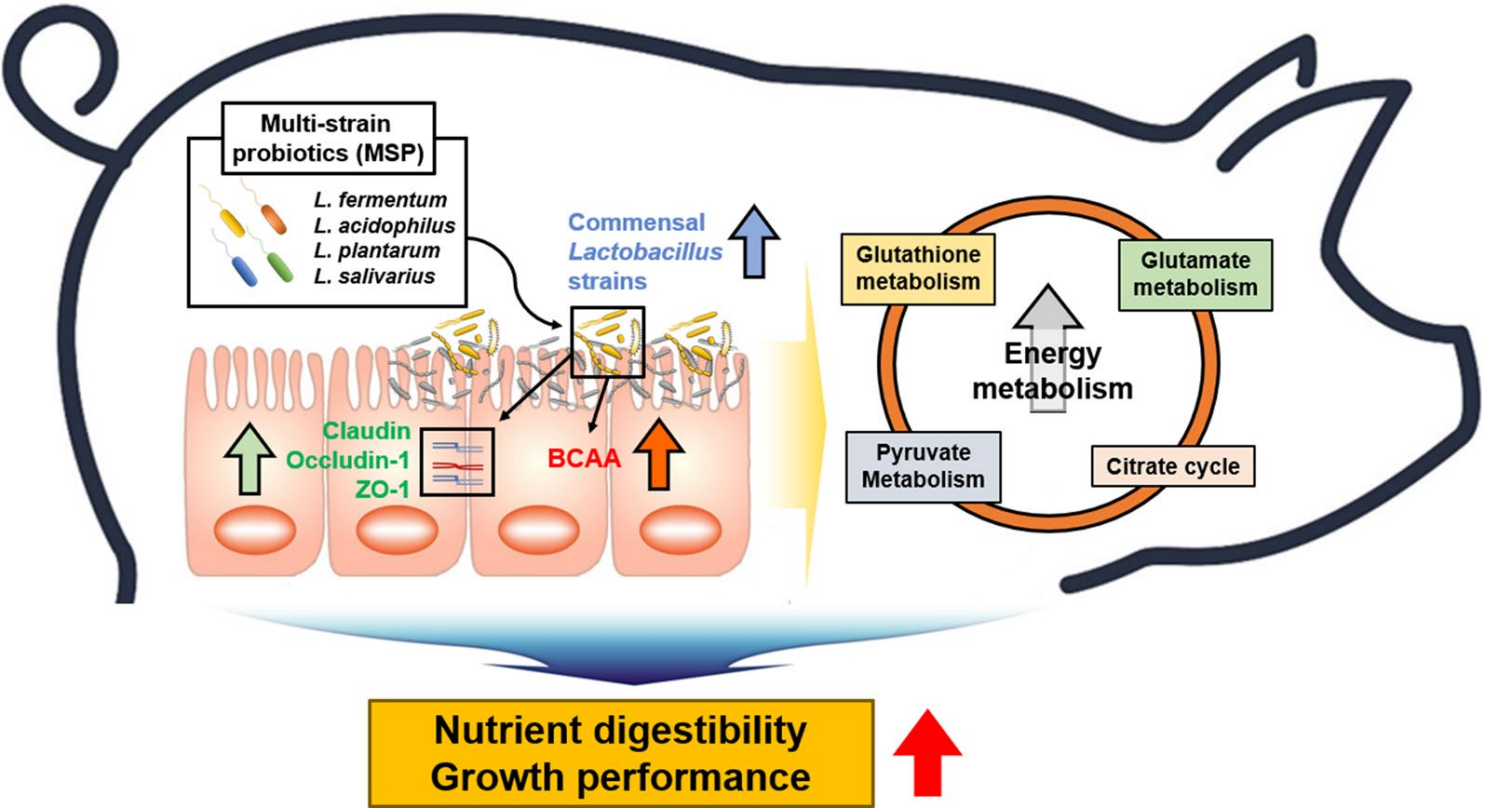
Schematic diagram. Schematic showing the results of integrated metagenomic, culturomics, and metabolomic analyses of the beneficial roles of MSP in weaning pigs. MSP increased the relative abundance of commensal *Lactobacillus* strains as well as improvement of BCAA production and energy metabolism. Furthermore, enhanced concentration of BCAA coupled with intestinal mucosal barrier integrity are considered to benefit piglets digest nutrients more efficiently, resulting in improved growth performance

To investigate the effects of commensal *Lactobacillus* which were increased by MSP supplement to piglets. 7 *Lactobacillus* isolated by culturomic approaches was conducted to investigate the functional effect to piglets in vitro. First, to evaluate whether commensal *Lactobacillus* enriched by MSP supplement were engaged with BCAA and energy metabolism, supernatants of commensal *Lactobacillus* strains were analyzed by GC-MS. Among supernatants samples from 7 *Lactobacillus*, *L. reuteri* increased BCAA including valine and isoleucine. Citric acid and pyruvic acid were increased by *L. ruminis*, *L. delbrueckii* subsp. *bulgaricus*, *L. sakei*, and *L. curvatus* in accordance with the previous result of MSP supplement increased energy metabolism (Fig. 7A, B). Moreover, to evaluate the substantial effect of *Lactobacillus* enriched by MSP supplement, the 7 species of *Lactobacillus* were treated to porcine jejunal cell line, IPEC-J2 and RT-qPCR was performed to assess the tight junction. The representative tight junction genes including claudin and ZO-1 were significantly up-regulated by all *Lactobacillus* except *L. sakei*, while *L. reuteri* and *L. mucosae* specifically up-regulated occludin-1 (Fig. 7C–E). These results indicated that commensal *Lactobacillus* enriched by MSP supplement were related to BCAA and energy metabolism which could enhance nutrient digestibility and growth performance as well as gut barrier function (Fig. 8).

## Discussion

Piglets are sensitive to nutritional, psychological, and physiological stress during weaning due to changes in food and ambient circumstances, which could result in accelerating taxonomic and functional alterations in the gut microbiome [34–36]. The administration of probiotics to piglets has shown a mechanism for preventing pathogen adhesion to the intestinal barrier and modulating the immune system [12, 37, 38]. In this study, as MSP was supplied to piglets, an alteration in microbial composition in the intestinal microbiota was observed, as well as a significant increase in the relative abundance of *Lactobacillus* when compared to control group. *Lactobacillus* exopolysaccharides last a long time in the intestine and promote *Lactobacillus* colonization, relieving lactose intolerance, modulating defense against infections [39]. Moreover, according to previous studies multi-strain probiotics supplement was tend to increased other species of *Lactobacillus* in gut microbiota [40, 41]. Likewise, our result revealed the supplement MSP influenced to elevate *Lactobacillus* in both metagenomic and culturomics analysis. The investigation of metabolites in swine feces revealed that higher quantities of acetate and butyrate that can be produced when fibre-fermenting *Lactobacillus* are present. These metabolites serve as a source of energy for the cells that make up the colon [42]. Weaned piglets fed *Lactobacillus* had a considerable increase in feed intake, which is a critical metric for reducing the burden of weaning [43, 44]. Further research discovered that the *Lactobacillus* group had a greater relative abundance of genes associated with metabolism of other amino acids, metabolism of terpenoids and polyketides, and biosynthesis of other secondary metabolites [45].

Culturomics has been established to cultivate and identify unknown bacteria by combining diverse culture conditions with fast identification [46]. Rather than seeing culture-dependent and independent techniques as a binary choice, the researchers suggest in a recent studies that both approaches complement one another [47]. Metagenomics is a powerful tool for determining community composition and gene content and requires less effort than culture-based approaches. As a result, recent research on the pig fecal microbiota has mostly concentrated on techniques that are independent on culture [48–50]. Metagenomic sequencing, on the other hand, is unable to determine the role of particular species in microbial communities. The value of culturomic-dependent methods has recently been demonstrated in human gut microbiome research, particularly in discovering processes that regulate bacterial populations [51, 52]. Culturomics techniques would considerably improve the understanding of how microbial communities develop and persist in pigs [22]. In this study, we suggest a technique for examining the microbiota of piglet feces. A total of 267 bacteria were isolated from the medium under various growth conditions, with *Lactobacillus*, *Escherichia*, and *Enterococcus* being the most prominent genera among the 42 species identified. In culturomics and metagenomics analysis, only *L. salivarius* was detected. However, 4 MSP *Lactobacillus* were revealed high adhesion ability to IPEC-J2 cell line compared with control *Lacticaseibacillus rhamnosus* strain GG, one of the most widely recognized probiotics that is extensively used in the market (Additional file 1: Fig. S2). This result revealed that though not all MSP *Lactobacillus* were detected in culturomics and metagenomics analysis, 4 MSP *Lactobacillus* are successfully capable to attach on the piglet intestine and influence on the dynamics of gut microbiota. Moreover, in metagenomics and culturomics analysis, 7 *Lactobacillus* species were enriched by MSP supplement.

MSP supplementation in piglets induced significant changes in functional KEGG pathways, especially BCAA. Valine and leucine are components of BCAA which play a crucial role in initiating catabolism. In particular, leucine has been considered as a stimulator of muscle-containing enzymes and branched-chain transferases. They are responsible for protein consumption; this is important since pig feed contains a high level of corn protein. However, excess levels of valine, leucine and leucine derivatives and isoleucine would be an occasion of imbalance in BCAA, and insufficient levels of BCAA could cause a decrease in growth performance [53, 54]. Supplementation with MSP upregulated both the biosynthesis and degradation of BCAA metabolism, which could be interpreted as MSP ascending the growth performance of piglets by triggering BCAA metabolism.

*Lactobacillus* enriched by MSP supplement up-regulated energy metabolism. When analyzed through metabolomic profile factors involved in citrate cycle such as glutathione, glutamate, pyruvate citrate and succinate was enhanced. Moreover, PPAR signaling pathway was also up-regulated by *Lactobacillus* enriched by MSP, which indicate the same context with previous studies. MSP metabolic pathway also denotes aminoacyl-tRNA synthetase (ARSs), which are “housekeepers” in the protein synthesis process, with the primary function of catalyzing the aminoacylation of transfer RNAs (tRNAs). According to previous findings, ARSs are involved in a wide range of physiological and pathological processes, such as angiogenesis, translation initiation, posttranslational modifications, autophagy and the anabolic insulin mechanism on muscle proteins [55, 56]. In addition, proline has been considered as one of the essential amino acids for piglet growth. Proline decreases the oxidation of phenylalanine, which limits the utilization of amino acids in inadequate dietary protein [57, 58], and uracil acts as an allosteric regulator and coenzyme processor in animals and plants, which aids in the production of various enzymes required for cell function by combining ribose and phosphates. Unfortunately, the specific use of uracil in pig metabolism has not yet been fully investigated, other than utilizing uracil as an excision DNA repair tool for viral disease studies [59]. However, previous studies demonstrated that uracil derivatives are competent supplements to fattening swine [60, 61]. On the basis of these findings, the increased level of uracil in response to MSP treatment could be interpreted as MSP supplementation being able to induce better development of piglets.

Our results indicated that the 7 *Lactobacillus* enhanced by MSP were beneficial to the host in both metabolomic and functional pathway analysis. Moreover, the previous study reported that BCAA promoted porcine intestinal β-defensins expression by activating the Sirt1/ERK/90RSK signaling pathway [62]. Which lead to, we hypothesized that BCAA, which were increased by commensal *Lactobacillus* enriched by MSP, could enhanced gut barrier function of piglets. Therefore, we investigated tight junction genes using IPEC-J2 cell line. *L. reuteri* and *L. mucosae* significantly increased expression of occludin-1, claudin and ZO-1, and other *Lactobacillus* also increased expression claudin, ZO-1. This result was resemble context of previous studies [63, 64], although precise pathway linking barrier function and amino acid was yet to be cleared, the result that supplement MSP influenced to *Lactobacillus* abundance of piglets fecal microbiota and increased commensal *Lactobacillus* strains elevated various metabolic pathways (especially energy metabolism) related to nutrient digestibility and growth performance of piglet could clarify (Fig. 8).

## Conclusion

Taken together, the MSP supplement diversified fecal microbiota by increasing the relative abundance of *Lactobacillus* of piglets, and MSP supplementation increased both BCAA and energy metabolism. Especially, an increase of commensal *Lactobacillus* abundance in the intestine is considered to benefit piglets digest nutrients more efficiently, resulting in improved growth performance. Notably, BCAA such as valine and isoleucine were increased in piglet fecal microbiota supplemented MSP and reproduced by *Lactobacillus* enriched by MSP. Moreover, *Lactobacillus* were able to enhance tight junction factors of IPEC-J2 cell line. However, this study requires research on these interactions in depth, still, we retained newly isolated 7 commensal *Lactobacillus* strains for facilitating further study.

## Methods

### MSP bacteria strains and farm-scale cultivation

Selected lactobacilli strains (*Lactobacillus acidophilus* LA14, *Ligilactobacillus salivarius* 167, *Lactiplantibacillus plantarum* LP11, and *Limosilactobacillus fermentum* JDFM 216) were used in this study for MSP application. These strains that selected for the large-scale production of MSP were isolated in a previous study for their potential probiotic characteristics [65, 66]. All lactobacilli (including *Lacticaseibacillus rhamnosus* strain GG as positive control in cell adhesion experiments) were cultured on de Man, Rogosa, and Sharp (Sigma–Aldrich, St Louis, MO, USA) with 0.05% l-cysteine (MRS-BCP) agar and incubated for 48 h at 37 °C. After ensuring the purity of colonies, the colonies were subcultured in MRS broth.

Next, after each strain were cultured in farm-scale medium (1% glucose, 1% molasses, 0.2% sea salt and 0.2% yeast extract) at 37 °C, they were combined and stored at 4 °C until further analysis for manufacturing MSP mixture as described previously [67]. For feed administration, MSP were mixed into a weaner diet every morning for MSP group (ca. 10^10^ CFU/kg of diet). The quantity of probiotics in the feed was measured once two weeks to ensure that MSP were supplemented effectively and appropriately.

### Experimental design, animals, diets, and sample collection

The procedure of experiment was reviewed and approved by the Institutional Animal Care and Use Committee of Chungnam National University, Daejeon, Republic of Korea (approval #CNU-00910). This study was carried out at the facility of Animal Research Center of Chungnam National University. In order to evaluate the beneficial roles with supplementation of MSP in weaning pigs using multi-omics analysis, a total of healthy 24 piglets (Landrace × Yorkshire × Duroc) were assigned to 2 treatments in a randomized complete block design (block = body weight [BW] and sex) with 4 pigs per pen and 3 replicated pens per treatment (Control group vs. MSP group). Both dietary treatments were formulated to meet or exceed the requirements of the National Research Council [68] of weaned pigs (Table 1). For 6 weeks (early stage with 3 weeks and late stage with additional 3 weeks), the control group continuously received the basal diet without the administration of antibiotics or probiotics, while the MSP group received the probiotics diet supplemented with MSP *ad libitum* with free access to fresh water. Pigs were housed in an environmentally controlled room and each pen was equipped with a feeder and water. For multiomics analysis, fresh fecal samples were collected from the rectum of each piglets in a 3 M sample bag (19 × 30 cm, 3 M, St, Paul, MN, USA) transported to the lab on ice quickly (within 30 min for culturomic approach) or stored at -80 °C until subsequent experiments (metagenomic and SCFA/metabolomic approaches). In addition, for direct plating counting, samples were homogenized and serially diluted. The diluted solution was plated on De Man-Rogosa-Sharpe (MRS; BD Difco, Sparks, MD, USA) agar for lactic acid bacteria, Bifidobacterium Selective (BS; KisanBio, Seoul, Korea) agar for *Bifidobacterium* spp., and Violet Red Bile Agar (Fisher scientific, Hampton, NH, USA) for coliforms and incubated at 37 °C for 48 h. Moreover, total aerobic bacteria and yeast/mold were evaluated on AC petrifilm (3 M, St, Paul, MN, USA) with cultivation at 37 °C for 48 h and YM petrifilm (3 M) with incubation at 25 °C for 72 h, respectively.

**Table 1:** Composition of basal diet of weaned pigs (as-fed basis)

Item	Phase I (Early stage)	Phase II (Late stage)
Ingredient, %		
Corn, 8%	51.68	48.55
Soybean meal, 44%	24.00	31.68
Wheat bran	10.00	8.00
Soybean hulls	7.40	7.50
Soybean oil	3.48	2.00
Monocalcium phosphate	1.90	1.15
Limestone	0.80	0.80
Vitamin-Mineral premix^a^	0.20	0.20
l-lysine-HCl	0.54	0.12
Calculated energy and nutrient contents		
Digestible energy, Mcal/kg	3.70	3.45
Crude protein, %	17.50	20.00
Digestible crude protein, %	14.00	15.00
Lysine, %	1.30	1.10
Calcium, %	0.70	0.60
Phosphorus, %	0.80	0.60
Crude fat, %	6.00	4.50
Crude fiber, %	4.00	6.00
Crude ash, %	6.00	5.00

^a^Vitamin–mineral premix provided the following quantities of vitamin–mineral per kilogram of basal diet: vitamin A, 12,000 IU; vitamin D3, 2500 IU; vitamin E, 30 IU; vitamin K3, 3 mg; D-pantothenic acid, 15 mg; nicotinic acid, 40 mg; choline, 400 mg; vitamin B12, 12 µg; Fe, 90 mg from iron sulfate; Cu, 8.8 mg from copper sulfate; Zn, 100 mg from zinc oxide; Mn, 54 mg from manganese oxide; I, 0.35 mg from potassium iodide; Se, 0.30 mg from sodium selenite

### DNA extraction, sequencing, and metagenome analysis for fecal samples in piglets

DNA was extracted from fecal samples using a PowerSoil DNA Isolation kit (MO BIO Laboratories, Carlsbad, CA, USA) according to the manufacturer’s protocol and previously described procedure [69] with slight modifications. Fecal samples were homogenized by vortexing for 2 min with sterile zirconia beads (0.1 mm zirconia; BioSpec, Cat. No. 11079101z). DNA concentration and quality were determined by measuring absorbance at 230, 260, and 280 nm using a spectrophotometer (SpectraMax ABS Plus, Molecular Devices, San Jose, CA). To amplify the V4 region from the 16S rRNA gene (primer set: forward, 5′-TCG TCG GCA GCG TCA GAT GTG TAT AAG AGA CAG GTG CCA GCM GCC GCG GTA A-3′; reverse, 5′-GTC TCG TGG GCT CGG AGA TGT GTA TAA GAG ACA GGG ACT ACH VGG GTW TCT AAT-3′) were used. 16 S rRNA gene amplicon sequencing was performed on the Illumina MiSeq platform at Macrogen, Inc. (Seoul, Republic of Korea). Samples with low Phred quality score (< Q30) were excluded from further analysis.

Fastq files obtained from MiSeq paired-end sequencing data were analyzed using Mothur (v. 1.41) [70]. The Mothur pipeline was used to process the entire sequence data according to the Mothur SOP stated manual. Briefly, error removal is performed through a nonaligned screening sequence with the Silva database [71] to include rare sequences in one or two base pairs to merge rare sequences into large sequences. Chimeric sequences were removed by using vsearch [72]. Taxonomic classification was analyzed using Greengenes-formatted databases [73] released in 2013, thereby eliminating sequences that are not categorized as archaea and bacteria. Singletons were removed using the Mothur subroutine “split.abund” [74], and operational taxonomic units (OTUs) were classified as distance 0.03 calculations (97% sequence similarity).

### Culturomic approaches to microbiological composition in piglets

The sample was collected in a 15 mL conical tube, and 1 g fecal sample with 9 mL of 0.85% NaCl buffer at a ratio of 1:9 (W/V, V/V) was mixed well by vortexing. After vortexing, the solution was serially diluted in anaerobic conditions by a Coy Anaerobic Chamber (Coy LAB Products, Grass Lake, Ml 49,240, USA) and spreading selective agar plates including (1) Fastidious Anaerobe Agar (NEOGEN, Lansing Ml 48,912, USA) with 7% Sheep Blood, (2) Phenylethyl Alcohol Agar (KisanBio) with 5% Sheep Blood; (3) MRS with 0.05% l-cysteine hydrochloride (Sigma-Aldrich, MO, St Louis, USA) Agar, (4) Bifidobacterium Selective (BS) (KisanBio) agar, and (5) Bacto™ Brain Heart Infusion broth (Fisher scientific) with 0.5% Difco™ Yeast Extract (Fisher scientific) and 10% Rumen’s fluid, and incubated at 37 °C for 72 °C. After incubation, 20 ~ 30 colonies of each agar plate medium were picked into new agar plate medium and then incubated for 72 h at 37 °C. For isolated pure single colonies, after incubation, colonies were substreaked and incubated under the same conditions, and this process was repeated again. After incubation, isolated bacteria in agar plate medium were stored in 10% skim milk (KisanBio, Seoul, Secho, Korea) at -81 °C. In addition, for isolation of endospore-forming bacteria under anaerobic conditions, samples were collected in 15 mL conical tubes, and 1 g fecal sample with 9 mL of 0.85% NaCl solution at a ratio of 1:9 (W/V, V/V) was mixed well by vortexing. After vortexing, the solution was treated with absolute ethanol (Merck, Burlington, MA, USA) at a ratio of 1:1 (W/V, V/V) at RT under anaerobic conditions for 4 h. After treatment, the mixture is centrifuged to remove ethanol, and the pellet was serially diluted under anaerobic conditions, spreading on Difco™ LB agar, and incubating for 72 h at 30 °C.

All isolated bacterial colonies (n = 267) were identified using 16 S ribosomal RNA sequencing. Sequencing results were analyzed by the NCBI BLAST algorithm for homologous sequence searches with type strains. Unclassified strains were defined as those with less than < 97% identity. If the identity of 16 S rRNA is within the range (> 97%), similarity has already been reported in the same genus.

### Short-chain fatty acid (SCFA) profiling

Sample preparation was carried out with modifications for HPLC–MS/MS analysis [75]. A total of 250 mg of pooled stool material was weighed and mixed with UltraPure™ water (Fisher Scientific, Hampton, NH, USA), vortexed, and centrifuged at 16,000 g for 10 min at 4°C. The supernatant was collected and mixed in a 1:1 ratio with methanol. The supernatant mixture was filtered through 0.2 µm pore size polyvinylidene fluoride (PVDF) syringe filters (Whatman, Maidstone, England). before subjected to HPLC–MS/MS analysis. The analysis method refers to [76] with modification. To analyze the MS/MS fragmentation conditions of three labeled SCFAs (3-nitrophenylhydrazine (Sigma–Aldrich, MO, St Louis, USA), N-(3-dimethylaminopropyl); 3-NPH, N-(3-dimethylaminopropyl)-N′-ethylcarbodiimide hydrochloride (Sigma–Aldrich, MO, St Louis, USA); EDC, VOA mixture (New Haven, CT, USA) in HPLC–MS/MS and separate HPLC, the derivatization method was used as follows. Prepare 100 µL sample solution that has been dissolved in 50% acetonitrile. Then, 100 mM 3-NPH 50 µL and 50 µL of 100 mM EDC were added, followed by incubation at 40 °C for 1 h. After incubation, 0.1% formic acid in 50% acetonitrile 100 µL and 50% acetonitrile 2 mL were added, and HPLC–MS/MS analysis was performed. HPLC–MS/MS analysis was performed using an integrated system composed of Nexera X2 (Shimadzu, Japan) and LCMS-8050 (Shimadzu, Japan). Then, 1 µL of reaction mixture of labeled SCFAs was injected into INNO C_18_ 3.5 μm, 250 × 2.1 mm (Youngjin Biochrom, Seongnam, Korea). Solvent A comprised water containing 0.1% formic acid, while solvent B was 100% acetonitrile. At a flow rate of 0.3 mL/min, labeled SCFAs were separated on an analytical column. The LC gradient method was set as follows: t = 0 min 80% A, 20% B; 0.5~5 min, 100% B; 0.5~5 min. All gradient methods were prosecuted gradually and held for 3 min at different gradients. The autosampler temperature was 40 °C, and the data were analyzed using an ESI source.

### Fecal metabolome analysis

The fecal samples were collected and stored at -80 °C until metabolomic analysis. Each fecal sample was weighed out and diluted in methanol to a final concentration of 20 mg/ml with vortexing for 5 min on ice. After centrifugation at 15,000*g* for 5 min at 4 °C, the upper layer of the supernatant was filtered with a 0.2 μm pore size polyvinylidene fluoride (PVDF) syringe filter. Aliquots of 200 µL of the filtered supernatant were concentrated to dryness in a vacuum concentrator and then stored at – 81 °C prior to derivatization and analysis by GC–MS. The extract was derivatized with 30 µL of a solution of 20 mg/mL methoxyamine hydrochloride in pyridine (Sigma, St. Louis, MO, USA) at 30 °C for 90 min, and 50 µL of N,O-bis(trimethylsilyl)trifluoroacetamide (BSTFA; Sigma) was subsequently added at 60 ℃ or 30 min. Fluoranthene was added to the extract as an internal standard. GC–MS analysis was conducted using a Thermo Trace 1310 GC (Waltham, MA, USA) coupled to a Thermo ISQ LT single quadrupole mass spectrometer (Waltham, MA, USA). A DB-5MS column with 60-m length, 0.2- mm i.d. and 0.25-µm film thickness (Agilent, Santa Clara, CA, USA) was used for separation. For analysis, the sample was injected at 300 °C and a split ratio of 1:60 with 90 mL/min helium split flow. The metabolites were separated with 1.5 mL constant flow helium with an oven ramp of 50 °C (2 min hold) to 180 °C (8 min hold) at 5 °C/min, to 210 °C at 2.5 °C/min, and to 325 °C (10 min hold) at 5 °C/min. The mass spectra were acquired in a scan range of 35–650 m/z at an acquisition rate of 5 spectra per sec. The ionization mode was subjected to electron impact, and the temperature for the ion source was set to 270 °C. The spectra were processed by Thermo Xcalibur software using automated peak detection, and the metabolites were identified by matching the mass spectra and retention indices of the NIST Mass spectral search program (version 2.0, Gaithersburg, MD, USA). The metabolite data were then normalized based on the intensity of the fluoranthene internal standard.

### Correlation analysis between metagenome and metabolome

For the microbial diversity analysis, R statistical software (version 3.6.3) was utilized. A nonparametric one-way analysis of variance (Kruskal–Wallis test) was used to analyze bacterial alpha diversities (Shannon, Chao index), followed by Tukey’s post hoc analysis if a significant difference (*p* < 0.05) was observed. To estimate distances for UniFrac, we used the phyloseq package [77] and then the vegan package [78] to conduct Adonis and ANOSIM tests to divide the variance across groups [79]. The variations in the relative abundance of bacterial composition were compared using Welch’s* t* test. In the M^2^IA server (http://m2ia.met-bioinformatics.cn/) [80], differential abundance of microbial communities at the genus level, as well as pairwise microbiome-metadata correlation analyses, were carried out. The correlation between the functional metabolites and bacteria was analyzed as a network. The pink nodes represent the functional metabolites and the other nodes of bacteria were grouped with different colors (functional false discovery rate (FDR) < 0.05). Significant correlations (p < 0.05) of functional metabolites and bacteria were plotted using a plugin of Cytocape (http://cytoscape.org) with the filter of MetScape3.1.3.

### IPEC-J2 cell line culture

Porcine intestinal epithelial cell line-J2 (IPEC-J2) was cultured in DMEM/F12 supplemented with 10% fetal bovine serum, 100 UI/mL penicillin-streptomycin, and 1x ITS–X (Gibco, Carlsbad, CA, USA) under 37 ℃ humidified environment with 5% CO_2_. The medium was replaced every other day according to the standard culture protocol. For experiments, cells were used between passage 17 and 25 and seeded on six-well plates with the concentration of 2.0 × 10^5^ cells/cm^2^. The selected 7 *Lactobacillus* species grown in MRS medium were washed with PBS (Sigma-Aldrich) for 5 times, resuspended in cell medium and treated to IPEC-J2 at the final concentration of 1.5 × 10^7^/mL. The cell medium was replaced with DMEM/F12 without antibiotic supplement in advance to bacterial treatment and the mixture was incubated at 37 ℃, 5% CO_2_ for 6 h. After the incubation, the medium with bacteria was removed. Cells were washed with PBS for 5 times and collected to extract total RNA. All experiments were conducted in triplicate.

### Cell adhesion ability assay

For cell adhesion experiments, IPEC-J2 cells were seeded on 24-well plates with the concentration of 2.4 × 10^5^ cells/cm^2^. The 4 MSP *Lactobacillus* species grown in MRS medium were washed with PBS for 5 times, resuspended in cell medium and treated to IPEC-J2 at the final concentration of 1.5 × 10^7^/mL. And then to evaluate the adhesion ability IPEC-J2 cell was incubated at 37 ℃ humidified environment with 5% CO_2_ for 2 h. To harvest IPEC-J2 cell, 200 µL trypsin-EDTA solution (Sigma-Aldrich) was treated to IPEC-J2 cell and incubated in 37 ℃ humidified environment with 5% CO_2_ for 10 min. After incubation, the cell was harvested and serial dilution with of 0.85% NaCl for CFU counting.

### RNA isolation and RT-qPCR analysis

Total RNA was extracted using RNeasy plus kit (Qiagen, Hilden, Germany) according to the manufacturer’s instruction and the quantity and quality of extracted RNA were measured using SpectraMax ABS Plus spectrometer (Molecular Devices, San Jose, CA, USA). Then, every total RNA sample was diluted to 1 ug and used to run one-step real-time PCR using Luna Universal Probe One-Step RT-qPCR Kit (New England BioLabs, Ipswich, MA, USA) with CFX96™ System (Bio-rad, Hercules, CA, USA). Each gene expression was normalized to the housekeeping gene, β-actin (^∆∆^CT) and the primer sequences used for this experiment were listed in Additional file 1: Table S1.

### Statistical analysis

Statistical analysis was performed using ANOVA. Tukey’s HSD test and Kruskal’s test were used for post hoc tests of significance using the R stats package. Calculations for Shannon, nonparametric Shannon diversity and Chao1 richness indices were performed using mothur version 1.41 [70]. UniFrac distance was analyzed to assess differences among sites based on phylogenetic information [79]. The statistical significance of the spatial structure of nonmetric multidimensional scaling (NMDS) plots was calculated using a UniFrac distance-based analysis of molecular variance (PERMANOVA). To identify functional pathways in the fecal microbiome, PICRUSt and Kyoto Encyclopedia of Genes and Genomes (KEGG) (level 2) were used to predict the presence of functional genes in the sample. PICRUSt [81] was conducted through an online application (https://huttenhower.sph.harvard.edu/galaxy/). The relative abundance difference of bacteria and significantly different KEGG pathways within groups were estimated using STAMP v0.2.1.3 [82] and an extended error bar plot.

## Supplementary Information

**Additional file 1: Fig. S1.** Relative abundance and diversity of fecal microbiota in MSP and control groups according to growth stage. (A) Relative abundance and diversity of fecal microbiota on the early- (at 3 weeks) and late-stage (at 6 weeks) in MSP group, (B) Relative abundance and diversity of fecal microbiota on the early- and late-stage in control group. Violin plots reflect differences in bacterial diversity in fecal microbiota according to the Shannon index and Chao richness. **Fig. S2.** Adhesion ability of 4 MSP *Lactobacillus* species. Adhesion ability of 4 MSP *Lactobacillus* species in IPEC-J2 cell line. LGG (*Lacticaseibacillus rhamnosus* GG) was employed as positive control. **Table S1.** Primers used for RT-qPCR.

## Data Availability

The datasets generated and/or analyzed during this study are available in the BioProject repository, http://www.ncbi.nlm.nih.gov/bioproject/830638. Additional datasets generated and/or analyzed during the current study are available from the corresponding author upon reasonable request.
