# Supplementary material for: Molecular characterization of gut microbiome in weaning pigs supplemented with multi-strain probiotics using metagenomic, culturomic, and metabolomic approaches
Source: Anim Microbiome. 2022 Nov 24;4:60. doi: 10.1186/s42523-022-00212-w (PMC9700986; doi:10.1186/s42523-022-00212-w)
Supplement: Supplementary file 1 — Additional file 1: Fig. S1. Relative abundance and diversity of fecal microbiota in MSP and control groups according to growth stage. (A) Relative abundance and diversity of fecal microbiota on the early- (at 3 weeks) and late-stage (at 6 weeks) in MSP group, (B) Relative abundance and diversity of fecal microbiota on the early- and late-stage in control group. Violin plots reflect differences in bacterial diversity in fecal microbiota according to the Shannon index and Chao richness. Fig. S2. Adhesion ability of 4 MSP Lactobacillus species. Adhesion ability of 4 MSP Lactobacillus species in IPEC-J2 cell line. LGG (Lacticaseibacillus rhamnosus GG) was employed as positive control. Table S1. Primers used for RT-qPCR. [file 42523_2022_212_MOESM1_ESM.docx]

**Supplementary material**

**Molecular characterization of gut microbiome in a weaning pig supplemented with multi-strain probiotics using metagenomic, culturomic, and metabolomic approaches**

Woong Ji Lee^1†^, Sangdon Ryu^1†^, An Na Kang^1^, Minho Song^2^, Minhye Shin^3^, Sangnam Oh^4*^, and Younghoon Kim^1*^

^1^Department of Agricultural Biotechnology and Research Institute of Agriculture and Life Science, Seoul National University, Seoul 08826, Korea

^2^Division of Animal and Dairy Science, Chungnam National University, Daejeon 34134, Korea

^3^Department of Microbiology, College of Medicine, Inha University, Incheon 22212, Korea

^4^Department of Functional Food and Biotechnology, Jeonju University, Jeonju 55069, Korea

^†^These authors contributed equally to this study.

^*^To whom correspondence should be addressed: osangnam@jj.ac.kr and ykeys2584@snu.ac.kr


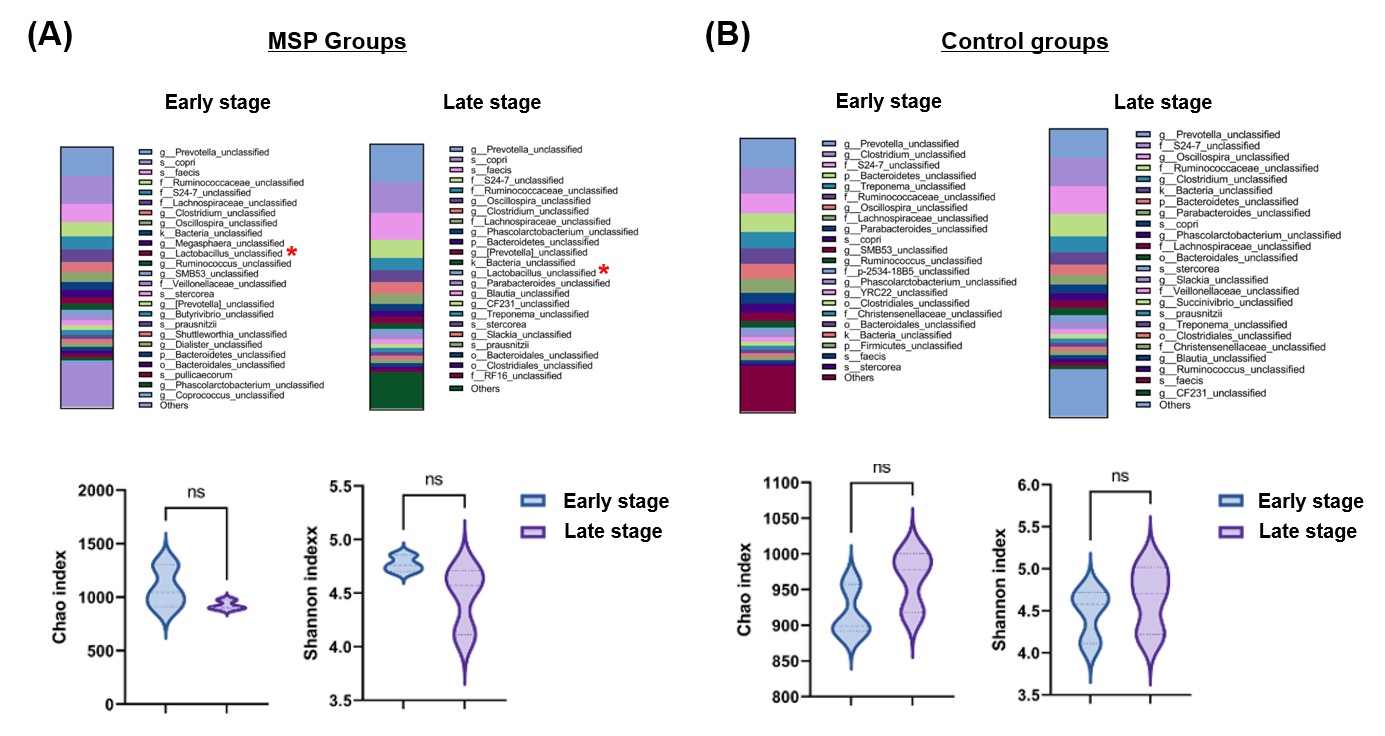


**Supplementary Figure S1.** Relative abundance and diversity of gut microbiota in MSP and control groups according to growth stage. (A) Relative abundance and diversity of gut microbiota on the early- (at 3 weeks) and late-stage (at 6 weeks) in MSP group, (B) Relative abundance and diversity of gut microbiota on the early- and late-stage in control group. Violin plots reflect differences in bacterial diversity in gut microbiota according to the Shannon index and Chao richness.

**Supplement Figure S2. Adhesion ability of 4 MSP *Lactobacillus* species.**  Adhesion ability of 4 MSP *Lactobacillus* species in IPEC-J2 cell line. LGG (*Lacticaseibacillus rhamnosus* GG) was employed as positive control.

**Supplementary Table S1.** Primers used for RT-qPCR

| **Genes** | **Sequence** | **bp** |
| --- | --- | --- |
| Claudin-1 | f: 5’-GCAGCAGCTTCTTGCTTCTC-3’ | 664 |
|  | r: 5’-CTGGCATTGACTGGGGTCAT-3’ |  |
| Occludin | f: 5’-ATCAACAAAGGCAACTCT-3’ | 157 |
|  | r: 5’-GCAGCAGCCATGTACTCT-3’ |  |
| ZO-1 | f: 5’-GAGTTTGATAGTGGCGTT-3’ | 298 |
|  | r: 5’-GTGGGAGGATGCTGTTGT-3’ |  |
| β-actin | f: 5’-TGCGGGACATCAAGGAGAAG-3’ | 216 |
|  | r: 5’-AGTTGAAGGTGGTCTCGTGG-3’ |  |
